# Gene-Related Cerebellar Neurodegeneration in SCA3/MJD: A Case-Controlled Imaging-Genetic Study

**DOI:** 10.3389/fneur.2019.01025

**Published:** 2019-09-24

**Authors:** Huirong Peng, Xiaochun Liang, Zhe Long, Zhao Chen, Yuting Shi, Kun Xia, Li Meng, Beisha Tang, Rong Qiu, Hong Jiang

**Affiliations:** ^1^Department of Neurology, Xiangya Hospital, Central South University, Changsha, China; ^2^Department of Neurology, Second Xiangya Hospital, Central South University, Changsha, China; ^3^Laboratory of Medical Genetics, Central South University, Changsha, China; ^4^Department of Radiology, Xiangya Hospital, Central South University, Changsha, China; ^5^National Clinical Research Center for Geriatric Diseases, Central South University, Changsha, China; ^6^Key Laboratory of Hunan Province in Neurodegenerative Disorders, Central South University, Changsha, China; ^7^Parkinson's Disease Center, Beijing Institute for Brain Disorders, Beijing, China; ^8^Collaborative Innovation Center for Brain Science, Shanghai, China; ^9^Collaborative Innovation Center for Genetics and Development, Shanghai, China; ^10^School of Information Science and Engineering, Central South University, Changsha, China; ^11^Department of Neurology, Xinjiang Medical University, Urumchi, China

**Keywords:** spinocerebellar ataxia 3, gray matter, white matter, ^1^HMRS, imaging genetics study

## Abstract

**Background:** Spinocerebellar ataxia type 3/Machado-Joseph disease (SCA3/MJD) is one of the nine polyglutamine (polyQ) diseases and is caused by a CAG repeat expansion within the coding sequence of the *ATXN3* gene. Few multimodal imaging analyses of the macro- and micro-structural changes have been performed.

**Methods:** In the present study, we recruited 31 genetically-confirmed symptomatic SCA3/MJD patients and 31 healthy subjects as controls for a multimodal neuroimaging study using structural magnetic resonance imaging (sMRI), proton magnetic resonance spectroscopy (^1^H-MRS) and diffusion tensor imaging (DTI).

**Results:** The SCA3/MJD patients displayed a significantly reduced of gray matter volume in the cerebellum, pons, midbrain and medulla, as well as inferior frontal gyrus and insula, and left superior frontal gyrus. The total International Cooperative Ataxia Rating Scale (ICARS) score was inversely correlated with the gray matter volume in the cerebellar culmen, pons and midbrain. The numbers of CAG repeats in the expanded alleles were inversely correlated with the gray matter in the cerebellar culmen. NAA/Cr and NAA/Cho ratio in the middle cerebellar peduncles, dentate nucleus, cerebellar vermis, and thalamus in the SCA3/MJD patients were significantly reduced when compared to that in the normal controls, suggesting neurochemical alterations in cerebellum in the SCA3/MJD patients. Tract-Based Spatial Statistics (TBSS) analysis revealed significant lower volume and mean FA values of the cerebellar peduncles, which inversely correlated with the total scores of ICARS in our patients.

**Conclusions:** In this study, we demonstrated cerebellar degeneration in SCA3/MJD based on tissue volume, neurochemistry, and tissue microstructure. Moreover, the associations between the clinical measures, cerebellar degeneration and genetic variation support a distinct genotype-phenotype relationship in SCA3/MJD.

## Introduction

Spinocerebellar ataxia (SCA) is a group of autosomal dominant neurodegenerative disorders with obviously clinical and genetic heterogeneity that is characterized by progressive loss of balance and coordination (1). To date, spinocerebellar ataxia type 3/Machado-Joseph disease (SCA3/MJD) is the most common SCA subtypes with an approximate frequency of 62.64% in mainland China (2–4). SCA3/MJD is caused by an unstable and expanded (CAG)_n_ trinucleotide repeats within the coding region of the *ATXN3* genes (5, 6). The CAG trinucleotide repeats range from 52 to 91 repeats in the SCA3/MJD patients, and <45 repeats in the normal people (7, 8). The neurological manifestations of SCA3/MJD include cerebellar ataxia, as well as pyramidal and extrapyramidal signs, nystagmus, dysarthria, and peripheral neuropathy etc. (9).

So far, neuroimaging study has shown widespread degeneration in CNS of SCA3/MJD patients, including the pons, cerebellar vermis and hemispheres, basal ganglia, midbrain, medulla oblongata, and cerebral cortex (10). Furthermore, MRI analysis of gray matter indicated that atrophy in the pons and vermis is especially obvious in SCA3/MJD patients, as well as the white matter surrounding the dentate nucleus and in the cerebellar peduncles (11). In addition, glucose utilization deficits in cerebellum, brainstem, and cerebral cortex can be observed in SCA3/MJD carriers even in the pre-symptomatic (12).

Although many studies on macrostructural atrophy in cerebral regions of SCA3/MJD patients have been performed, there was little understanding of the changes in microstructure and the relationship between the clinical and genetic assessments (11, 13). In this study, we performed a cross-sectional study of multimodal imaging, including sMRI, ^1^HMRS, and DT-MRI in SCA3/MJD patients to investigate the changes of the microstructure underlying the neurodegenerative process.

## Materials and Methods

### Subjects

**Figure 1:**
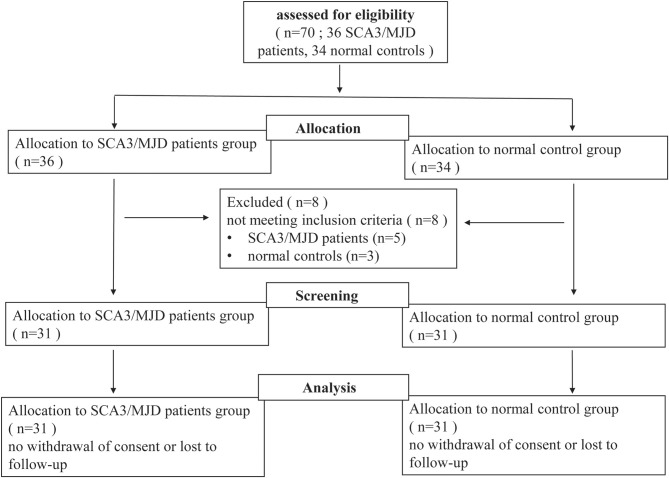
Flow-chart of the study. Seventy participants (including 36 SCA3/MJD patients and 34 normal controls) volunteered and were screened for inclusion and exclusion criteria. Five SCA3/MJD patients had to be excluded because they were younger than 18 years old. Three normal controls had to be excluded because they had neurological or psychiatric deficits. Sixty-two participants (including 31 SCA3/MJD patients and 31 normal controls) were enrolled in the study.

In this study, 31 SCA3/MJD patients, which were diagnosed with Harding criteria, were recruited from the Neurodegenerative Disorders Clinic of the Departments of Neurology of the Xiangya Hospital of Central South University in the People's Republic of China. In this exploratory study, patient recruitment depended on their willingness to give informed consent and the availability of MRI scanning and genetic test. Furthermore, each patient was evaluated according to a standardized clinical examination procedure that includes systematic physical and neurological examinations. The ataxia severity was assessed by using the ICARS (14). The number of CAG repeats was calculated by genetic analysis of expanded allele in *ATXN3* gene. 31 controls that were sex- and age-matched to the patients were recruited from the community. Exclusion criteria for all participants were as follows: (1) younger than 18 years old; (2) pregnancy or breastfeed; (3) neurological diseases, psychiatric deficits, metabolic diseases and tumors; (4) any contraindications for MRI examination. The workflow of recruitment was illustrated in Figure 1.

### Images Acquisition

Images acquisition was performed using a Siemens Sonata 1.5-tesla MRI scanner (Siemens Medical Systems, Erlangen, Germany) at Xiangya Hospital Imaging Center.

First, high-resolution whole-brain T1-weighted images were obtained using the MPRAGE sequence with the following parameters: TR/TE = 1,900/4.38 ms, flip angle = 30°C and isotropic voxel size 1 × 1 × 1 mm^3^.

**Figure 2:**
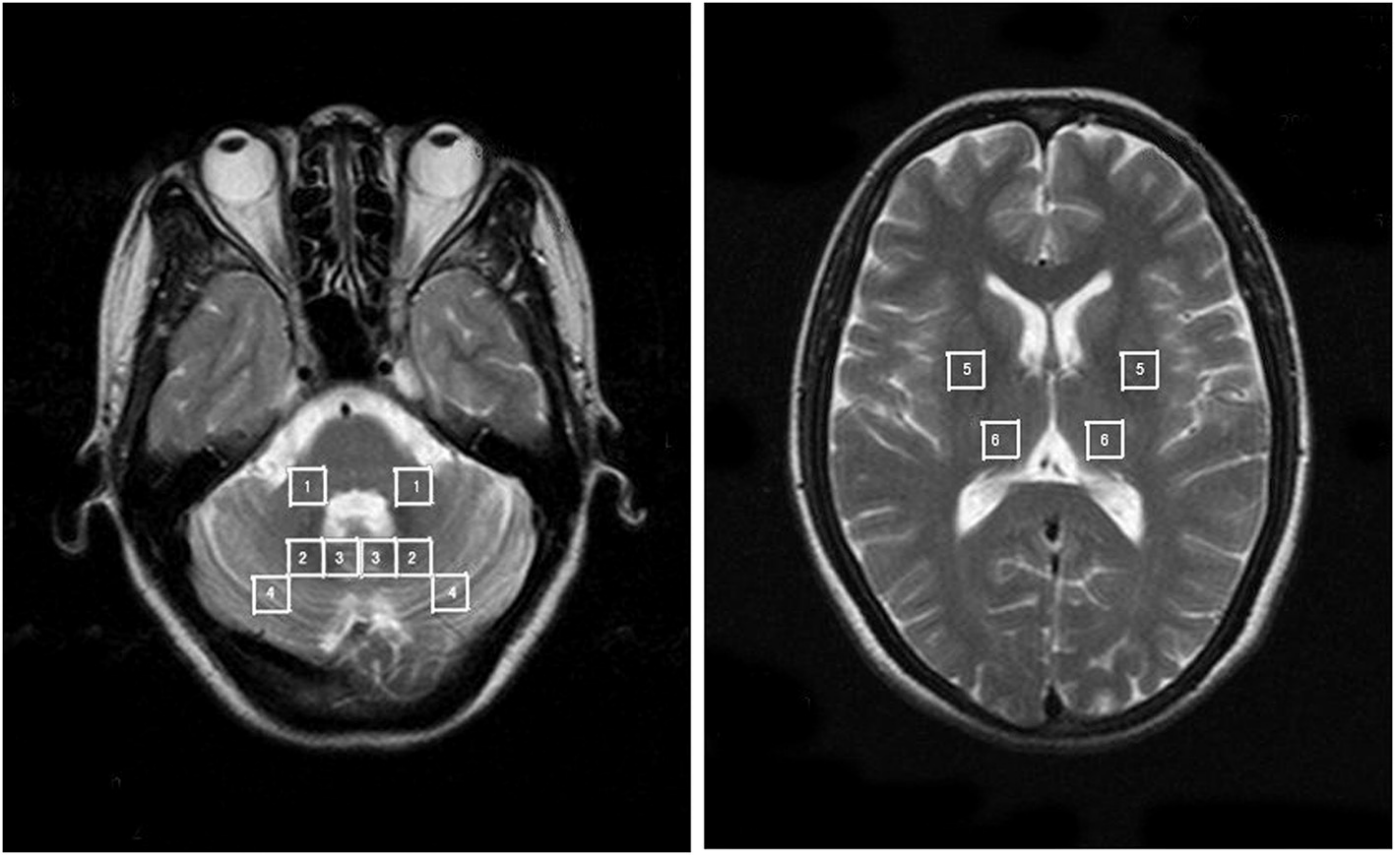
The anatomically defined ROIs in the ^1^HMRS images. 1 = MCP; 2 = dentate nucleus; 3 = cerebellar vermis; 4 = cerebellar cortex; 5 = putamen; 6 = thalamus.

Second, 3 dimensional T1-weighted images (TR/TE = 450/10 ms, field of view = 26 cm, matrix = 256 × 256, 5 mm thickness and 1.5 mm gap for axial images, 6 mm thickness and 1.5 mm gap for sagital and coronal images) and axial T2-weighted images (TR/TE = 4,200/98 ms, field of view = 24 cm, matrix = 256 × 256, 5 mm thickness and 1.5 mm gap) of the whole brain were acquired via spin-echo and fast spin-echo sequences, respectively. Then, ^1^HMRS data were acquired using a standard PRESS sequence (TR/TE = 1,500/135 ms) from two axial slices, which were paralleled to the anterior commissure-posterior commissure (AC-PC) plane (Figure 2). The first slice that included the cerebellum and the pons was defined by referring to the midpoint of the pons in the sagittal view of the T1 images. The second plane was defined across the basal ganglia and thalamus. ^1^HMRS data were obtained from four regions of interest (ROIs) (i.e., the middle cerebellar peduncle, dentate nucleus, cerebellar vermis, and cerebellar cortex) in the first slice and two ROIs in the second slice. Each ROI was examined bilaterally for the presence of lateralization.

Third, diffusion weighted images along the AC-PC plane were acquired via a single-shot echo planar imaging sequence using the following parameters: TR/TE = 9,900/99 ms, field of view = 256 × 256 mm^2^, matrix = 128 × 128, slice thickness = 2 mm and 60 continuous axial slices without a gap. The diffusion sensitizing gradients were applied to 12 non-linear directions (b = 1,000 s/mm^2^), together with an acquisition image without diffusion weighting (b = 0 s/mm^2^).

### Data Processing and Statistical Analysis

#### sMRI Images Analysis

Voxel-based morphometry (VBM) was performed to explore the difference in gray matter intensity between the SCA3/MJD patients and controls. VBM analysis was performed using the Statistical Parametric Mapping (SPM5) software (http://www.fil.ion.ucl.ac.uk/spm/) according to the following steps. (1) After removing the scalp tissue, skull, and dural venous sinus voxels, the brain was segmented into gray matter, white matter and cerebrospinal fluid partitions (in native space). (2) The gray matter partitions were spatially normalized using a 12-parameter affine transformation and 7 × 8 × 8 basis functions. The normalized gray images were averaged and smoothed, respectively, by applying a Gaussian kernel of 8 mm full width at half maximum (FWHM) to generate customized gray matter templates. (3) The deformation parameters resulting from the above normalization were applied to the original whole-brain images to produce optimally normalized images, which were then segmented into gray matter images using the unified segmentation model (15) followed by a hidden Markov random field model clean-up step. To increase classification accuracy, we adapted no priors option during segmentation to avoid deviation of the tissue in our samples from the ICBM gray matter priors, which were generated from a sample with a mean age of 25 years. (4) A Jacobian modulation step was applied to the segmented images to preserve the volume of gray matter within each voxel. The modulated images were then smoothed using a kernel with a FWHM of 10 mm for further analysis.

The two-sample *t*-test within the General Linear Model in SPM5 was used to evaluate the difference in gray matter intensity between the SCA3/MJD patient and control groups. Moreover, the associations between the gray matter volume and the total ICARS scores or the numbers of CAG repeats in expanded alleles of SCA3/MJD patients were evaluated via a multiple regression using a threshold of *p* < 0.05 (FDR corrected).

#### ^1^H-MRS Image Analysis

^1^HMRS image preprocessing included zero-filling, Gaussian apodization, Fourier transformation, water reference processing, frequency shift correction, and phase and baseline correction using Functool software (2.6.4b version). The spectral quality was reflected by the FWHM in parts-per-million of the proton frequency of 63.8 MHz. Spectra of poor quality were discarded prior to statistical analysis. The peak integral values were determined by using the curve-fitting software provided by the manufacturer. NAA was assigned to be 2.02 ppm, Cho was assigned to be 3.2 ppm, and Cr assigned to be 3.02 ppm. The NAA/Cr and NAA/Cho ratios were calculated from voxels in the aforementioned six pairs of ROIs.

Metabolic group differences for each ROI between groups were evaluated via the independent-samples *t-*test. Pearson correlation was used to determine the associations between the metabolic indices for each ROI and the total ICARS scores or the numbers of CAG repeats in the expanded alleles in the SCA3/MJD patients group. *P*-values were Bonferroni corrected for six tests.

#### DTI-MRI Image Analysis

First, the difference in the FA between the two groups were evaluated using the voxel-based approach. For each subject, the unweighted diffusion image (b = 0) was normalized first normalized to the EPI template of the SPM5 in standard Montreal Neurological Institute (MNI) space. This normalization consists of a 12 degree-of-freedom linear transformation and a non-linear transformation using 7 × 8 × 7 basis functions. Then, the transformation parameters were applied to normalize the FA image into standard MNI space. Finally, the normalized FA images were spatially smoothed using an 8 × 8 × 8-mm^3^ FWHM Gaussian kernel (16). The resulting image was superimposed onto the average normalized FA images of all subjects for visualization.

**Figure 3:**
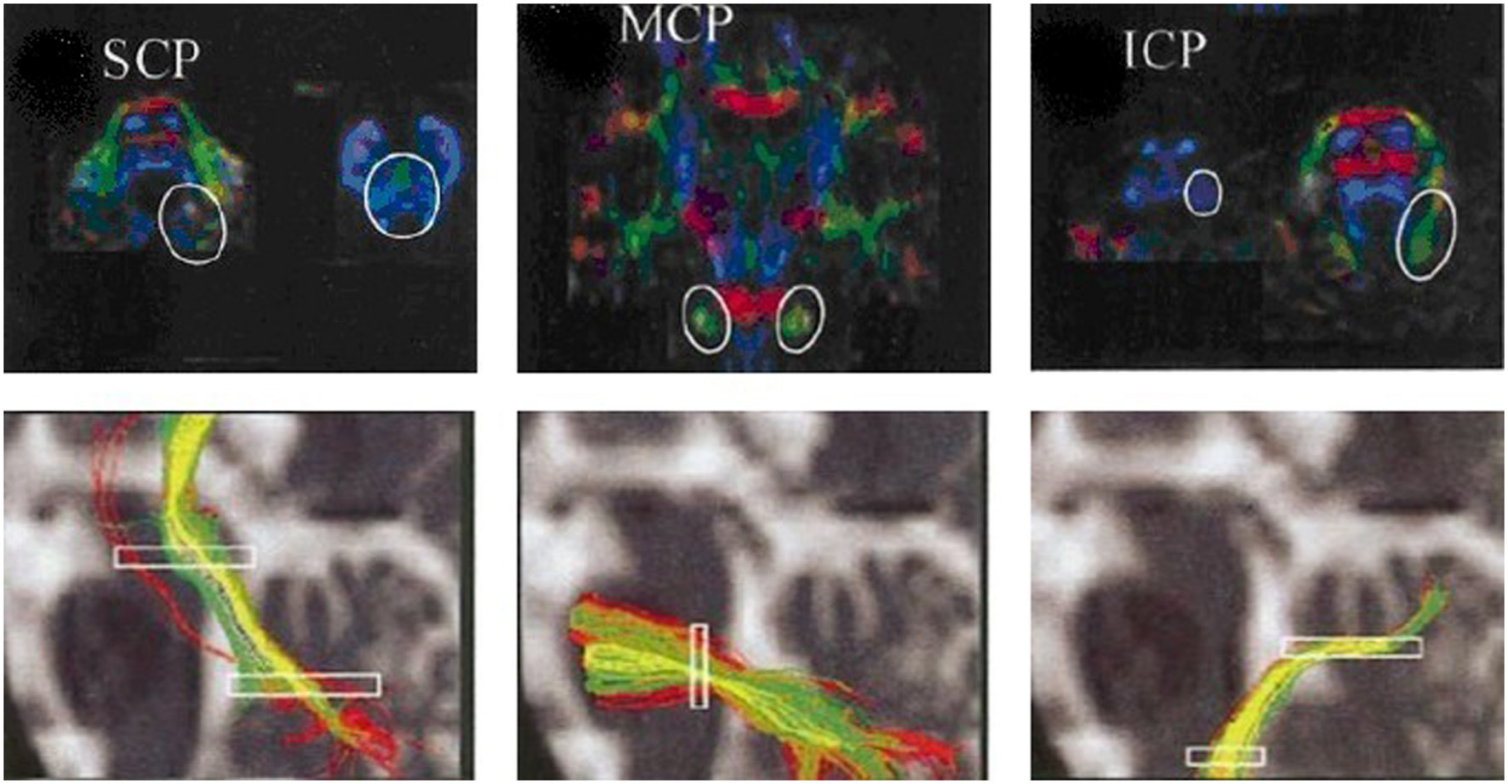
ROIs of the cerebellar peduncles in the color-coded FA maps. The anatomically defined ROIs of the cerebellar peduncles in the color-coded FA maps (superior row) and their resulting fiber tracts (inferior row). SCP, superior cerebellar peduncle; MCP, middle cerebellar peduncle; ICP, inferior cerebellar peduncle.

Second, tractography was performed using the DTI-studio software (Version 2.40) (Johns Hopkins University). The superior cerebellar peduncles, middle cerebellar peduncles and inferior cerebellar peduncles (SCP, MCP, and ICP, respectively) were reconstructed individually for controls based on the “fiber assignment by continuous tracking” method (17). All of the fiber tracts were reconstructed using voxels FA values >0.1. Tractography was terminated at an angle >50° or at voxel FA value <0.1. The SCP, MCP, and ICP were defined according to ROI-based tractography in the directionally color-coded FA images according to anatomical knowledge (18) (Figure 3). Fibers passing through two anatomically selected ROIs were regarded as the corresponding fiber tracts. The SCP was determined based on the two ROIs of the cerebellum and the midbrain; the two MCP was determined based on the ROIs of the left and right lateral pontine tegmentum; and ICP was determined based on the ROIs of the medullar and cerebellum. Because of the patients' cerebellar peduncles were less reliably identified, we used the probability maps of the cerebellar peduncles of the controls group to calculate the diffusion indices of the corresponding fiber tracts in the patient group.

The SCP probability map was obtained as follows: First, the b = 0 images were normalized into the standard MNI space, and the voxels were resampled as 2 × 2 × 2 mm^3^. The transformation parameters were then applied to the coordinates of the curves forming the fiber bundles. After the three-dimensional SCP mask was created via ROI-based tractography, a SCP probability map was obtained by averaging the SCP masks of all controls. The value of each voxel in the probability map was regarded as the probability that the voxel was part of the SCP. After the SCP probability map was generated, the indices of the SCP were calculated according to following procedure. The b = 0 image was normalized for each subject, and the resulting transformation parameters were applied to normalize the FA images, the indices of the SCP were obtained by superimposing the probability map on the normalized diffusion images. The FA and MD values of the MCP and the ICP for each subject were obtained in the same manner.

Two-sample *t*-tests were performed to evaluate the differences in the FA values of the cerebellar peduncles between the two groups. Pearson correlations were used to evaluate the associations between the FA values of the cerebellar peduncles and total ICARS scores or the numbers of CAG repeats in expanded alleles of the SCA3/MJD patients. *P-*values were Bonferroni corrected for three tests.

## Results

### VBM Analysis Between SCA3/MJD Patients and Normal Control

**Figure 4:**
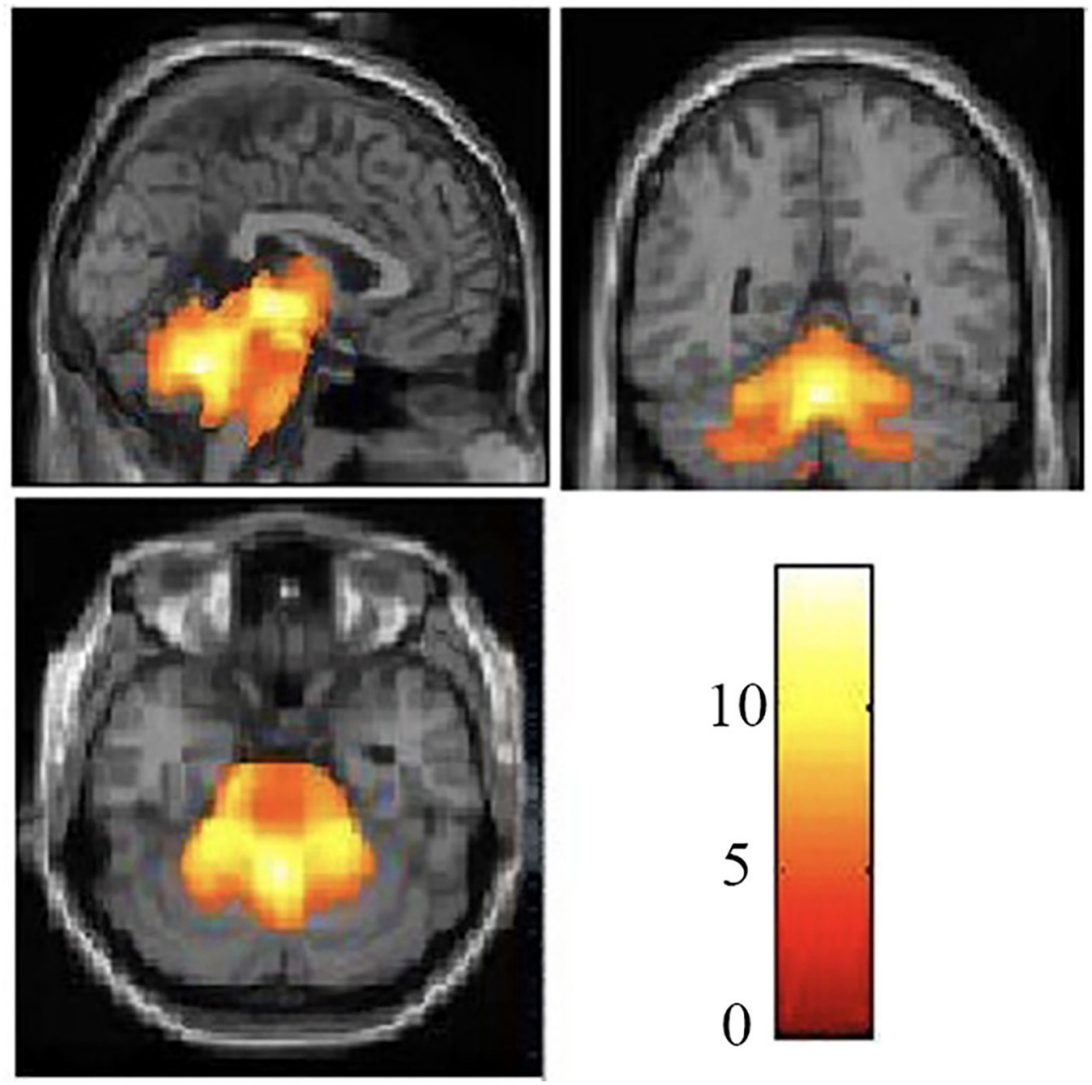
Reduced white matter volume in the SCA3/MJD patients. Reduced gray matter volume in the SCA3/MJD patients compared with the controls based on voxel-based morphometry (*p* < 0.05). The color bar refers to the *T*-values.

The demographic and clinical characteristics of the participants are presented in Table 1. There was no significant difference in age or gender between the SCA3/MJD patients and the controls (*p* > 0.05). Compared with the controls, the SCA3/MJD patients displayed a significant reduction in gray matter volume in the cerebellum, pons, midbrain and medulla, as well as inferior frontal gyrus and insula, and left superior frontal gyrus (*p* < 0.05, FDR corrected) (Figure 4, Table 2).

**Table 1:** Demography and clinical assessments of the controls and SCA/MJD subjects.

	**Controls**	**SCA3/MJD patients**
Sex (male/female)	15/16	15/16
Age (year)^a^	37.53 ± 10.18	38.91 ± 7.38
Age of onset (year)^a^	NA	34.88 ± 6.64
Duration of disease (year) ^a^	NA	4.81 ± 3.63
ICARS total score^a^	NA	26.81 ± 10.82
CAG trinucleotide repeats length ^a^	NA	71.84 ± 2.61

a *Expressed as mean ± SD. NA, not applicable*.

**Table 2:** Reduced gray matter volume in the SCA3/MJD patients via the voxel-based morphometry.

**Regions**		**Cluster size**	***T*-value**	**Centroid voxel**		
**Lobes**	**Labels**			**x**	**y**	**z**
Cerebellum	Right cerebellar culmen	171,811	14.40	0	−51	−27
	Left cerebellar culmen		12.53	−13	−16	−22
Sub lobar	Left Insula	1,051	5.37	−34	−23	9
	Right Insula	542	4.24	40	−5	9
Frontal lobe	Left Inferior frontal gyrus	337	4.56	−36	25	−19
	Right Inferior frontal gyrus	144	4.40	22	19	−21
	Left Superior frontal gyrus	352	4.70	−5	52	37

Moreover, correlation analysis of the SCA3/MJD patients group revealed that the total ICARS score and disease duration were inversely correlated to the gray matter volume of cerebellar culmen, pons, and midbrain (*p* < 0.005), and the number of CAG repeats in expanded alleles was inversely correlated to the gray matter volume of cerebellar culmen (*p* < 0.01) (Table 3).

**Table 3:** The ICARS total scores or CAG was correlated with the gray matter volume in the SCA3/MJD patients.

**Indices**	**Gray matter regions**	**Cluster size**	***T*-value**	**MNI coordinate (x, y, z)**		
ICARS	Left cerebellar culmen	246	4.64	−5	62	−9
	Right pons	1,215	4.02	14	−26	−40
	Left midbrain	264	3.67	−2	−42	−21
	Left cerebellar lingual			−2	−42	−15
CAG	Right cerebellar culmen	215	2.89	25	−42	−22

### ^1^H-MRS Analysis Between SCA3/MJD Patients and Normal Control

We found that the values of NAA/Cr and NAA/Cho ratio were significantly reduced in the MCP, dentate nucleus, cerebellar vermis, and thalamus in the SCA3/MJD patients compared to that in the normal controls (*p* < 0.001, MCP; *p* < 0.001, dentate nucleus; *p* < 0.001, cerebellar vermis), whereas only NAA/Cr ratio in cerebellar cortex was significantly decreased (*p* < 0.01, cerebellar cortex). There was a significant difference of Cho/Cr ratio in cerebellar vermis between SCA3/MJD patients and controls (*p* < 0.01). In addition, no significant difference of NAA/Cr, Cho/Cr, and NAA/Cho ratio were observed in the putamen between the SCA3/MJD group and the control group (Table 4).

**Table 4:** Neurochemistry of the ^1^HMRS images in the controls and patient group.

**Regions of interest**	**Metabolic ratios**	**Controls (mean ± SD)**	**SCA3/MJD patients (mean ± SD)**	***P*-value**
Middle cerebellar peduncle	NAA/Cr	2.21 ± 0.59	1.41 ± 0.44	*P* = 0.00^*^
	Cho/Cr	1.18 ± 0.29	1.10 ± 0.34	*P* = 0.22
	NAA/Cho	1.91 ± 0.41	1.31 ± 0.36	*P* = 0.00^*^
Dentate nucleus	NAA/Cr	1.32 ± 0.24	1.07 ± 0.22	*P* = 0.00^*^
	Cho/Cr	1.07 ± 0.18	1.01 ± 0.20	*P* = 0.06
	NAA/Cho	1.25 ± 0.20	1.07 ± 0.19	*P* = 0.00^*^
Cerebellar vermis	NAA/Cr	1.22 ± 0.23	0.96 ± 0.25	*P* = 0.00^*^
	Cho/Cr	1.00 ± 0.18	0.88 ± 0.23	*P* = 0.002^*^
	NAA/Cho	1.24 ± 0.23	1.12 ± 0.28	*P* = 0.01
Cerebellar cortex	NAA/Cr	1.17 ± 0.33	1.02 ± 0.27	*P* = 0.008^*^
	Cho/Cr	0.92 ± 0.24	0.88 ± 0.23	*P* = 0.37
	NAA/Cho	1.31 ± 0.34	1.23 ± 0.37	*P* = 0.23
Putamen	NAA/Cr	1.22 ± 0.27	1.23 ± 0.34	*P* = 0.81
	Cho/Cr	0.87 ± 0.30	0.95 ± 0.31	*P* = 0.16
	NAA/Cho	1.48 ± 0.44	1.37 ± 0.43	*P* = 0.15
Thalamus	NAA/Cr	1.68 ± 0.37	1.47 ± 0.47	*P* = 0.008^*^
	Cho/Cr	1.05 ± 0.29	0.99 ± 0.34	*P* = 0.18
	NAA/Cho	1.63 ± 0.35	1.47 ± 0.24	*P* = 0.003^*^

* *After Bonferroni adjustment for multiple testing, p ≤ 0.008 was considered as significant in the correlation analysis (six tests)*. *NAA, N-acetyl-aspartate; Ch, choline-containing compounds; Cr, creatine and phosphocreatine*.

In SCA3/MJD patients, the total of ICARS score and disease duration were inversely correlated with the NAA/Cr ratio in MCP and the dentate nucleus, as well as Cho/Cr ratio in the dentate nucleus, respectively. In addition, the longer disease durations in SCA3/MJD patients were associated with decreased Cho/Cr ratio in cerebellar vermis (Table 5). Furthermore, the NAA/Cr ratio in cerebellar cortex and the NAA/Cho ratio in the cerebellar vermis were inversely correlated to the length of CAG repeats in expanded alleles of the SCA3/MJD group (*r* = −0.400, *p* < 0.05; *r* = −0.409, *p* < 0.05, respectively).

**Table 5:** Relationship between ^1^H-MRS and clinic variable in SCA3/MJD group.

**Regions of interest**	**Metabolic ratios**	**ICARS (r)**	**Duration of disease (*r*)**
Middle cerebellar peduncle	NAA/Cr	−0.45^**^	−0.54^**^
	Cho/Cr	−0.27	−0.0.27
	NAA/Cho	−0.27	−0.23
Dentate nucleus	NAA/Cr	−0.50^**^	−0.57^**^
	Cho/Cr	−0.37^**^	−0.51^**^
	NAA/Cho	−0.003	0.06
Cerebellar vermis	NAA/Cr	−0.28	−0.34
	Cho/Cr	−0.26	−0.45^**^
	NAA/Cho	0.01	0.14
Cerebellar cortex	NAA/Cr	0.01	−0.12
	Cho/Cr	0.14	−0.07
	NAA/Cho	0.05	−0.06
Putamen	NAA/Cr	−0.16	−0.18
	Cho/Cr	−0.22	−0.11
	NAA/Cho	0.09	−0.02
Thalamus	NAA/Cr	−0.18	0.04
	Cho/Cr	−0.20	−0.05
	NAA/Cho	−0.02	0.01

** *p < 0.001. After Bonferroni adjustment for multiple testing, p ≤ 0.008 was considered as significant in the correlation analysis (six tests)*.

*NAA, N-acetyl-aspartate; Ch, choline-containing compounds; Cr, creatine and phosphocreatine*.

### White Matter Differences Between SCA3/MJD Patients and Normal Control

**Figure 5:**
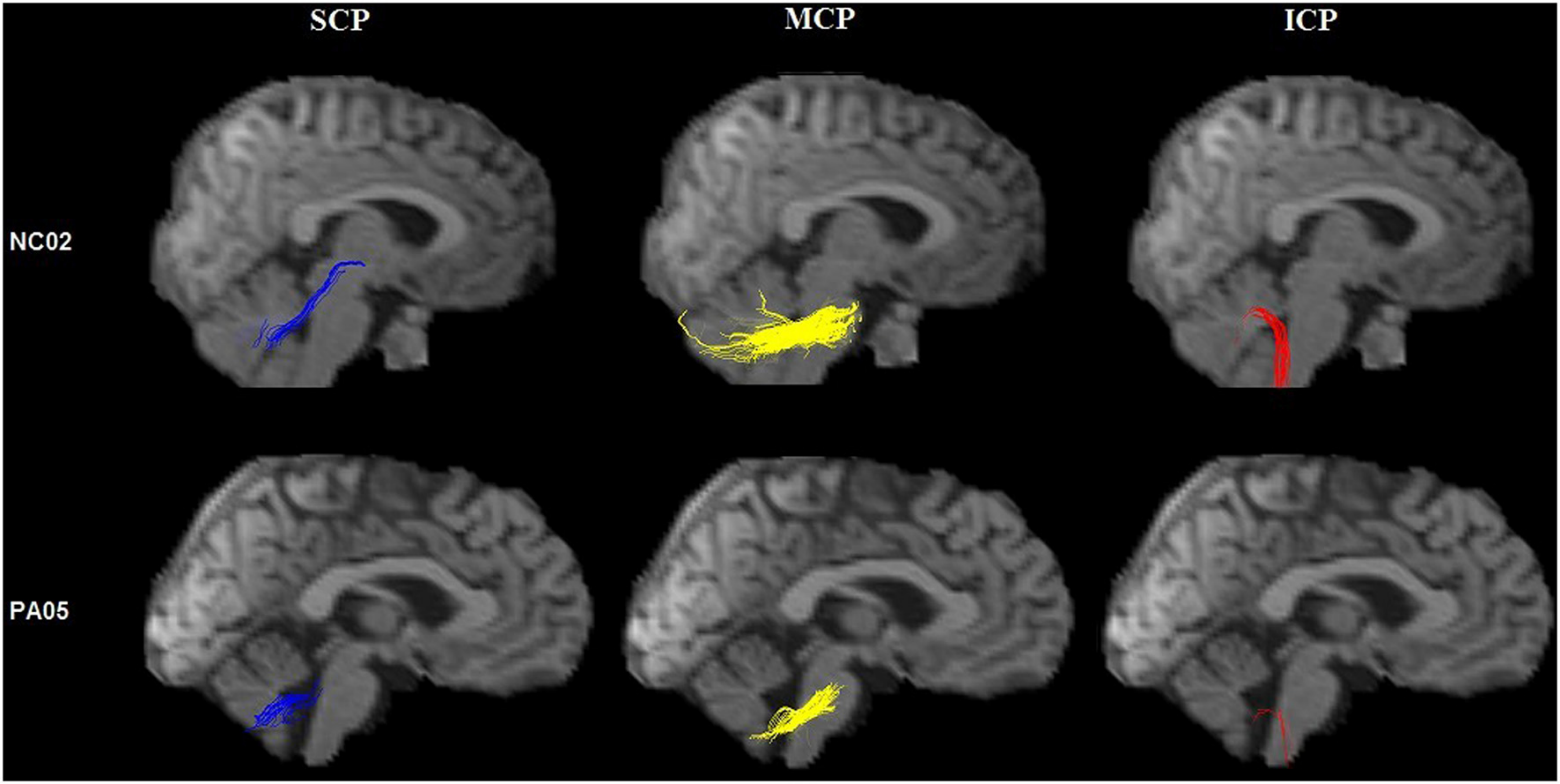
Tractography of the cerebellar peduncles. Tractography of the cerebellar peduncles in a control (upper row) and a SCA3/MJD patient (lower row).

TBSS analysis revealed significantly lower volume and mean FA values in the cerebellar peduncles of the SCA3/MJD patients (*p* < 0.001). Significant differences in FA and MD between SCA3/MJD patients and healthy controls were identified in the SCP, MCP, and ICP (*p* < 0.001) (Figure 5, Tables 6, 7). Furthermore, the correlation analysis was conducted between the FA/MD values and disease duration, the ICARS, and CAGs. The total ICARS score was inversely correlated to the FA values in all three cerebellar peduncles of the patients (SCP: *r* = −0.644, *p* < 0.001; MCP: *r* = −0.421, *p* < 0.05; ICP: *r* = −0.602, *p* < 0.001, respectively). However, the total ICARS score was inversely correlated with MD only in the SCP. Similarly, the disease duration was inversely correlated with FA in three cerebellar peduncles (*r* = −0.50~ −0.70, *p* < 0.001), and correlated with MD in the SCP (*r* = 0.63, *p* < 0.01) (Table 8). However, no significant correlation was found between the FA values in the three cerebellar peduncles and the numbers of CAG repeats in expanded alleles in our study.

**Table 6:** Reduced fractional anisotropy in the cerebellar peduncles of the SCA3/MJD patients.

**Regions of interest**	**Controls (mean ± SD)**	**SCA3/MJD patients (mean ± SD)**
Superior cerebellar peduncle	0.37 ± 0.02	0.28 ± 0.03^**^
Middle cerebellar peduncle	0.38 ± 0.03	0.29 ± 0.03^**^
Inferior cerebellar peduncle	0.34 ± 0.04	0.23 ± 0.03^**^

** *p < 0.0001. After Bonferroni adjustment for multiple testing, p ≤ 0.01 was considered as significant in the correlation analysis (three tests)*.

**Table 7:** Increase mean diffusivity in the cerebellar peduncles of the SCA3/MJD patients.

**Regions of interest**	**Controls (mean ± SD)**	**SCA3/MJD patients (mean ± SD)**
Superior cerebellar peduncle	0.74 ± 0.04	0.86 ± 0.07^**^
Middle cerebellar peduncle	0.69 ± 0.03	0.76 ± 0.06^**^
Inferior cerebellar peduncle	0.75 ± 0.09	0.92 ± 0.10^**^

** *p < 0.0001. After Bonferroni adjustment for multiple testing, p ≤ 0.01 was considered as significant in the correlation analysis (three tests)*.

**Table 8:** Relationship between FA and MD and clinic variable in SCA3/MJD group.

	**Superior cerebellar peduncle**		**Middle cerebellar peduncle**		**Inferior cerebellar peduncle**	
	**FA**	**MD**	**FA**	**MD**	**FA**	**MD**
ICARS	−0.64^**^	0.56^**^	−0.42*	0.44*	−0.60^**^	0.34
Duration	−0.68^**^	0.63^**^	−0.53^**^	0.43*	−0.69^**^	0.32

** *p < 0.001. After Bonferroni adjustment for multiple testing, p ≤ 0.01 was considered as significant in the correlation analysis (three tests)*.

## Discussion

The study revealed cerebellum and related region degenerations in Chinese SCA3/MJD patients by multimodal neuroimaging and also demonstrated their correlation between the degenerations and clinical measures as well as CAG abnormal expansions.

The cerebellum lesion was not the whole story, other regions were also involved. Interestingly, we observed significant gray matter volume loss in the frontal gyrus and insula and left superior frontal gyrus of SCA3/MJD patients, which is consistent with other studies showing that gray matter volume was not only significantly reduced in the pons and the vermis, but also in supratentorial regions including the frontal lobe, temporal lobe, parietal lobe, occipital lobe, putamen, and caudate (19, 20). One reason for the cortical area was involved is that structural lesion in SCA3/MJD begins in the spinal cord, cerebellar peduncles, as well as substantia nigra and progresses to cerebral areas in the long term (21). Another study reported that pallidal atrophy may be observed in SCA3/MJD patients with disease duration over 10 years (22). Thus, no obvious involvement of basal ganglia in our study might be attributed to relative short duration (mean ± SD, 4.81 ± 3.63).

In addition, we also observed significant lower volume and mean FA values in the cerebellar peduncles of the SCA3/MJD patients, which might indicate abnormal microstructure changes in these tracts, possibly providing new clues for pathological studies. The result was consistent with SCA3/MJD pathological studies that revealed neuron loss in the cerebellar dentate nucleus with myelin loss (23). Moreover, we also found that the decrease of NAA/Cr and NAA/Cho ratio in the MCP, dentate nucleus, cerebellar vermis, and thalamus in present study, suggesting SCA3/MJD mainly affected the middle cerebellar peduncle and dentate, where the degree of neuronal dysfunction accompanied by comparable cerebellar ataxia and disease duration. Many studies have showed a decrease in the NAA/Cr ratio or in the concentration of NAA in cerebellar regions in polyQ diseases, such as SCA1, SCA2, SCA3/MJD, and SCA6 (24, 25). These results indicated the atrophy of the cerebellum and brainstem was related to the predominant clinical features in SCA3/MJD patients. Gray matter and white matter were both involved, although the cerebellar nuclei may be the mainly involved region.

In this study, we demonstrated that there might be a correlation between the atrophy profile and clinical and genetic features (i.e., ICARS total score and the size of the abnormal CAG repeats lengths). Specifically, the volume changes in the cerebellar culmen, pons, and midbrain inversely correlated with the total ICARS score, suggesting that cerebellum and brainstem is related to predominant clinical features in SCA3/MJD patients. In addition, we also found an inverse correlation between FA values in the cerebellar peduncles and ICARS total score. The ICP mainly contains afferent fibers receiving information from movement centers, and a variety of sensory information related to movement. The MCP mainly is composed of fibers from the pons nucleus to the cerebellum. The SCP contains most of the efferent fibers projecting directly, or indirectly through the thalami, to the frontal cortex (26). There was significant degeneration in all three cerebellar peduncles in SCA3/MJD. The reduced white matter integrity in all three cerebellar peduncles were correlated with cerebellar ataxia symptoms and disease duration. Similar findings were also identified in other polyQ diseases, such as SCA1, SCA2, SCA7, and DRPLA (27–29), and revealed that white matter tract abnormalities in the whole brain across polyQ diseases, which is possibly owing to similar pathological mechanisms. Furthermore, we also found that FA values of SCP, MCP, and ICP had an inverse relationship with disease duration of SCA3/MJD. Previous study reported that white matter tracts across the whole brain were impaired in the asymptomatic stages of SCA3/MJD, and abnormal white matter tracts were closely related to SCA3/MJD disease severity, including movement disorder and cognitive dysfunction (30).

Additionally, in our cohort, we found the numbers of CAG repeats in expanded alleles were inversely correlated with the atrophy of the cerebellar culmen and cerebellar cortex. Some studies demonstrated significant correlation between the atrophy of brainstem, cerebellar, and tegmentum of pons and the CAG repeats length (31–33), whereas some failed to indicate the correlation between the CAG repeats expansion and the anatomical changes in the cerebellum or brainstem (34–36). The disease progression and ethnic difference might be the potential reasons for these controversial findings, suggesting further studies across with different regions with large cohort need to be implemented.

In terms of potential limitations, this study is a clinic-based and cross-sectional study, instead of a population-based and longitudinal study. In particular, the sample size is relatively small and the information of asymptomatic mutation carriers was not available, which makes it difficult to acquire a comprehensive understanding from asymptomatic to symptomatic status. On the other hand, the present study confirmed previous findings using multiple neuroimaging modalities.

In conclusion, this is an imaging-genetic study to explore the degeneration of cerebellar and its correlation with *ATXN3* gene in Chinese SCA3/MJD patients. The macrostructural and microstructural changes showed by reduction of gray matter volume, neurochemical alterations, and white matter degeneration in the brain of our patients provided converging evidence of neurodegeneration for SCA3/MJD, which supported the genotype-phenotype relationship in such disease.

## Data Availability Statement

The datasets generated for this study are available on request to the corresponding author.

## Ethics Statement

The present study was approved by the Ethical Committee of the Xiangya Hospital, Central South University. All subjects gave their written informed consent to participate the study.

## Author Contributions

HP and XL: study conception, design and organization, acquisition of data, analysis and interpretation of data, drafting of the manuscript, critical revision of the manuscript for important intellectual content, statistical analysis, administrative, technical, and material support. ZL, ZC, and YS: analysis and interpretation of data, critical revision of the manuscript for important intellectual content, and statistical analysis. LM, KX, and BT: drafting the work or revising it critically for important intellectual content. RQ and HJ: study conception, design and organization, analysis and interpretation of data, drafting of the manuscript, critical revision of the manuscript for important intellectual content, statistical analysis, administrative, technical, material support, and study supervision.

### Conflict of Interest

The authors declare that the research was conducted in the absence of any commercial or financial relationships that could be construed as a potential conflict of interest.
