# Corrigendum: Gene-Related Cerebellar Neurodegeneration in SCA3/MJD: A Case-Controlled Imaging-Genetic Study

**DOI:** 10.3389/fneur.2020.00030

**Published:** 2020-02-07

**Authors:** Huirong Peng, Xiaochun Liang, Zhe Long, Zhao Chen, Yuting Shi, Kun Xia, Li Meng, Beisha Tang, Rong Qiu, Hong Jiang

**Affiliations:** ^1^Department of Neurology, Xiangya Hospital, Central South University, Changsha, China; ^2^Department of Neurology, Second Xiangya Hospital, Central South University, Changsha, China; ^3^Laboratory of Medical Genetics, Central South University, Changsha, China; ^4^Department of Radiology, Xiangya Hospital, Central South University, Changsha, China; ^5^National Clinical Research Center for Geriatric Diseases, Central South University, Changsha, China; ^6^Key Laboratory of Hunan Province in Neurodegenerative Disorders, Central South University, Changsha, China; ^7^Parkinson's Disease Center, Beijing Institute for Brain Disorders, Beijing, China; ^8^Collaborative Innovation Center for Brain Science, Shanghai, China; ^9^Collaborative Innovation Center for Genetics and Development, Shanghai, China; ^10^School of Information Science and Engineering, Central South University, Changsha, China; ^11^Department of Neurology, Xinjiang Medical University, Urumchi, China

**Keywords:** spinocerebellar ataxia 3, gray matter, white matter, ^1^HMRS, imaging genetics study

There is an error in the Funding statement. The correct number for the National Natural Science Foundation of China is No. 81771231 to HJ and No. 81600995 to YS.

Additionally, we neglected to include the funder **the National Natural Science Foundation of China**, **No. 81974176 to HJ; No. 81901169 to ZC**. The corrected funding statement appears below.

## Funding

We declared that there were no financial interests in this study. This study was supported by the National Key Research and Development Program of China (Nos. 2016YFC0901504 and 2016YFC0905100 to HJ; No. 2016YFC1306000 to BT), the National Natural Science Foundation of China (Nos. 81771231 and 81974176 to HJ; No. 81600995 to YS; No. 81901169 to ZC), the National Natural Science Foundation of Hunan Province (No. 2019JJ40363 to RQ), Key Research and Development Program of Hunan Province (No. 2018SK2092 to HJ), Scientific Research Foundation of Health Commission of Hunan Province (No. B2019183 to HJ), The Clinical and rehabilitation fund of Peking University Weiming Biotech Group (No. xywm2015I10 to HJ).

The authors apologize for this error and state that this does not change the scientific conclusions of the article in any way. The original article has been updated.

